# Long-Term Remission of Acquired Von-Willebrand's Disease and Platelet Dysfunction after High-Dose Melphalan in a Patient with Multiple Myeloma

**Published:** 2019-01-01

**Authors:** Jan Stratmann, Stefan Gundermann, Christof Geisen, Alexandra Dukat, Wolfgang Miesbach

**Affiliations:** 1 Department of Hemostaseology, Johann Wolfgang Goethe University of Frankfurt, Frankfurt am Main, Germany; 2 German Red Cross Blood Donor Department, Frankfurt am Main, Germany; 3 Department of Hematology and Oncology, Johann Wolfgang Goethe University of Frankfurt, Frankfurt am Main, Germany

**Keywords:** Acquired von-Willebrand's disease, Multiple myeloma, Autologous stem cell transplantation, Platelet dysfunction, Immunoglobulin

## Abstract

**Background: **Autologous stem cell transplantation is considered a standard of care treatment in eligible patients with multiple myeloma, but puts the patient at high risk for infections and bleeding complications. Acquired von-Willebrand's disease (AVWD) and acquired platelet dysfunction are rare bleeding disorders that are associated with lymphoproliferative disorders such as multiple myeloma. Patients with acquired bleeding disorders who are planned for ASCT to treat the underlying condition are considered at highest risk for bleeding complications, and optimal treatment strategies are not known.

**Materials and Methods:** We summarized the diagnostic and therapeutic approach to a patient affected by AVWD and acquired platelet disorder related to multiple myeloma. The patient who was planned for ASCT presented with moderate to severe bleeding symptoms.

**Results:** Acute bleeding episodes were successfully controlled and prevented during induction and consolidation therapy with immunoglobulins, whereas replacement of plasma-derived VW factor showed no clinical improvement. High-dose melphalan-based consolidation therapy supported with autologous stem-cell transplantation led to an immediate and sustainable rise of von-Willebrand antigen and activity and a subsequent normalization of platelet aggregation activity. After a follow-up of 40 weeks, the patient maintained normalized VW levels and platelet aggregation capacity. There were no further signs or symptoms of bleeding.

**Conclusion**: This case report highlights the necessity for combined supportive and causal treatment in patients with AVWD and paraproteinemic PD. High-dose melphalan with autologous stem cell support may function as a treatment option in patients with myeloma-related AVWD.

## Introduction

 Multiple myeloma (MM) is a malignant plasma-cell disorder whose clinical features include lytic bone lesions, anemia, kidney failure and hypercalcemia. The initial therapy of symptomatic MM varies depending on risk stratification and fitness of the patient; however, international guidelines recommend high-dose chemotherapy followed by autologous stem cell transplantation (ASCT) in eligible patients to improve survival^1^. Patients who undergo ASCT are at high risk for infections and bleeding complications due to transient severe pancytopenia and prophylactic measures, including the use of broad spectrum antibiotics and transfusion of platelet and blood products^2^^,^^3^. 

Acquired von-Willebrand disease (AVWD) and acquired platelet dysfunction (APD) are rare bleeding disorders that are associated with lymphoproliferative disorders such as MM. Clinical features involve mucocutaneous bleeding, menorrhagia and prolonged bleeding from minor bruises or dental procedures, thus resembling the inherited VWD and inherited PD in clinical findings ^4^. 

Patients with acquired bleeding disorders who are planned for ASCT to treat the underlying condition are considered at high risk for bleeding complications. Optimal treatment strategies have not been established due to the limited number of affected patients and multiple pathophysiologic mechanisms of these acquired bleeding disorders^5^. In general, causal treatment of the underlying condition is considered crucial for sustainable response and supportive treatment (such as the administration of desmopressin, replacement of VWF, immunoglobulins, plasmapheresis and antifibrinolytics) aiming to control and prevent severe bleeding episodes^6^.

In the following case report, we describe a patient affected by AVWD and APD related to MM. We review our diagnostic workup and treatment approach to control acute bleeding events, prevent bleeding in high-risk situations and obtain long-term remission. Written informed consent was provided by the patient.

## Case presentation

A 62-year-old male patient with MM IgG kappa, ISS 1^7^, anemia (10.3g/dl [normal range 13.5 – 17-5g/dl]) and two isolated bone lesions in the spine was referred to the cancer center for treatment initiation (laboratory findings [standard values in parentheses]: β2-microglobulin, 3.2mg/l [0.8 – 2.4mg/l]; M-gradient, 0.26g/dl, 4% [0g/dl, 0%]; 90% bone marrow infiltration with plasmatic cells [0.5 – 3.0%]; serum free light chain ratio, 8.72 [0.26-1.65]; body weight: 65 kilogram). 

His medical history involved diabetes mellitus, atopic dermatitis and Bechterew´s disease with severe arthrosis of the right hip causing a wheelchair-dependency most time of the day. A noticeable bleeding history was documented: the patient suffered from nose bleeding since he was a child as well as intermittent severe gastrointestinal bleeding episodes due to angiodysplasia in the jejunum and ileum, which were controlled by local endoscopic interventions at accessible sites. However, an open appendectomy as a child and inguinal hernia repair two years ago were performed without major bleeding events. The family history for bleeding disorders was negative. There were no specific findings in the full body examination. 

The patient was referred to our department of hemostaseology for further diagnostic workup of the reported bleeding episodes in advance of the planned induction therapy for the MM. The coagulation tests showed VWF: Ag 12% [60 – 150%], VWF: Act < 4% [47,8 - 173,2%], VIII:C 13.6% [68 - 133%], aPTT 51s [25-37sec], bleeding time epinephrin>220sec [84 – 160sec], bleeding time ADP > 227sec [68-121sec]), a reduced platelet aggregation induced by ristocetin of 9% [89-100%] and ADP of 63% [88-100%] and normal levels of coagulation factor IX and XIII. Multimer analysis revealed absence of all proportions of VWF, compatible with type 3 VWD. Administration of VWF concentrate in combination with antifibrinolytics was recommended in case of acute bleeding. Presence of an inhibiting autoantibody against VWF was excluded in our and a reference laboratory. 

The patient was treated with four induction cycles of VCD (Bortezomib 1,3mg/m^2^ d1,4,8,11; Cyclophosphamid 900mg/m^2^ d1; Dexamethason 40mg d1-12) ^8^ and one cycle of CAD (cyclophosphamide 1g/m^2^ d1; adriamycin 15g/m^2^ d1-4; dexamethasone 40mg d1-4)^9^ for stem-cell mobilization, accompanied by standard of care supportive therapy. The patient suffered from two self-limiting mild bleeding events and low-grade polyneuropathy. A cumulative dose of 18250 units (95IE/kg bodyweight per day) of a plasma-derived FVIII/VWF combination concentrate (Haemate P) was administered on three consecutive days for continuous central-line insertion-site bleeding during stem cell collection. The patient achieved a stable disease after completion of the induction therapy.

**Fig. 1:**
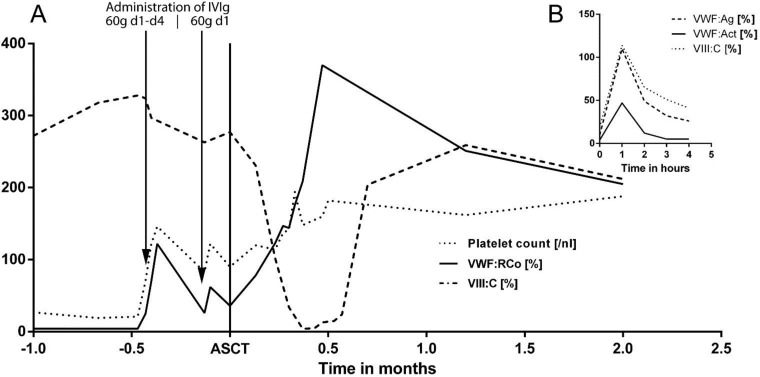
(A) Development of coagulation factors and platelets during treatment: VWF:Act, von Willebrand factor activity; VIII:C, factor VIII activity; IVIg, immunoglobulin; ASCT, d0 of consolidation therapy: autologous stem cell transplantation; (B) recovery of VWF:Ag, VWF:Act and VIII:C after administration of 92 units/kg bodyweight Haemate P (plasma-derived VWF / FVIII concentrate)

The patient was re-referred to our department in advance of the planned consolidation therapy for nose bleeding that was not manageable with local compression for more than 24hours. We administered an initial dose of 6000 units (92 units/kg bodyweight) of Haemate P, but low concurrent recovery values with less than 10% VWF: Act three hours after VWF administration (see Figure 1B) indicated a rapid clearance of VWF. We hypothesized a MM associated AVWD and administered immunoglobulines (IVIg) 1g per kg bodyweight for four consecutive days. A rapid recovery of VWF and reoccurrence of all proportions of VW multimers could be documented on day 2 after one dose of 60g IVIg. In line with the laboratory findings, the formerly uncontrolled nose bleeding stopped after the first IVIg dose. Administration was repeated once (60g IVIg) to maintain normal VWF levels throughout the consolidation therapy (Figure 1A). Impairment of platelet aggregation, however, was not ameliorated by IVIg administration. 

 Consolidation was performed with high-dose melphalan at a dose of 200 mg per square meter of body-surface area supported with autologous stem cell transplantation (ASCT) using 2.57 x 10^6^ CD34^+^ cells per kilogram bodyweight (melphalan 100mg/m^2^ d-3, d-2; stem cell support d0)^3^. The patient suffering from neutropenic fever was successfully treated with antibiotics and no bleeding events were identified. An immediate and sustainable rise of VWF and FVIII after ASCT was documented (Figure 1A). In addition, ristocetin-induced thrombocyte aggregation and in-vitro bleeding time normalized 5 weeks after ASCT. The patient achieved a partial remission according to the international response criteria evaluated 9 weeks after consolidation therapy^10^. Coagulation factors were normal at last follow-up, 40 weeks after ASCT. There were no clinical signs or symptoms of bleeding. 

## Discussion

 To our knowledge, this is the first report on a long-term remission of a patient diagnosed with AVWD and PD as a complication of MM after administration of high-dose melphalan supported with ASCT.

Distinguishing acquired from inherited VWD is challenging and anamnestic findings play a major role in making the diagnosis. In our case, the personal history including childhood bleeding events but negative family history was inconclusive, leading to the initial assumption of inherited VWD. In the course of disease, rapid clearance of plasma-derived VWF gave rise to the correct diagnosis. Repeatedly, autoantibodies against VWF could be excluded. The general frequency of inhibiting autoantibodies in AVWD is low, and therefore does not provide enough sensitivity for the diagnosis of AVWD^4^. 

Treatment of AVWD is challenging and aims to control acute bleeding events, prevent bleeding in high-risk situations like chemotherapy-induced pancytopenia and obtain long-term remission. Immunoglobulins are a reasonable therapy option for lymphoproliferative-related AVWD, especially in IgG-restricted MM^11^. All proportions of VW multimers could already be detected after the first IVIg administration of 60g (0.92g per kg bodyweight), leading to a prolongation of the VWF half-life from less than one hour (Fig. 1B) to a few days (Fig. 1A). 

Long-term remissions of AVWD using immunomodulatory drugs (e.g. thalidomide) and proteasome inhibitors (e.g. bortezomib) have been described^12^^,^^13^, but time to treatment response ranged between weeks and months in these cases, providing limited usefulness in patients with high clinical burden of AVWD. This is however the first case of a long-term remission of AVWD achieved by high-dose melphalan supported with ASCT. A sustainable rise of VWF could be documented from day one after reconstitution with autologous stem cells that reached a maximum VWF activity within 2 weeks after ASCT, a critical phase where therapy-related thrombocytopenia causes a high risk for spontaneous bleeding events (Fig. 1A). Rise of VWF was accompanied by a significant reduction of paraproteinemia, albeit level of paraprotein does not necessarily correlate with clinical AVWD severity or laboratory findings^14^. 

Of interest, IVIg was not able to restore platelet function, indicating a non-immunogenic impairment of ristocetin-induced platelet aggregation. In accordance to Djunic et al. who performed systematic mixing studies and showed that adding IVIg to the reaction mixture was not able to restore platelet activity in vitro^15^, we have provided the first corresponding clinical evidence that IVIg fails to significantly change platelet aggregation capacity in paraproteinemic impairment. Platelet function and in-vitro bleeding time however subsequently normalized after high-dose melphalan therapy, presumably due to the general reduction of paraprotein burden.

In summary, this case report highlights the necessity for close anamnestic review of the patient´s history as well as the need for combined supportive and causal treatment in patients with AVWD and paraproteinemic APD. Close monitoring and an interdisciplinary approach involving hematologists and hemostaseologists are of great importance to manage patients with these rare acquired bleeding disorders. Immunoglobulins provide short-term usefulness to bridge patients who are at high-risk for bleeding complications. High-dose chemotherapy followed by ASCT can be considered as a salvage treatment option for patients suffering from these rare bleeding disorders. 

## References

[1] Moreau P, San Miguel J, Ludwig H (2013). Multiple myeloma: ESMO Clinical Practice Guidelines for diagnosis, treatment and follow-up. Ann Oncol..

[2] Fermand JP, Ravaud P, Chevret S (1998). High-Dose Therapy and Autologous Peripheral Blood Stem Cell Transplantation in Multiple Myeloma: Up-front or Rescue Treatment? Results of a Multicenter Sequential Randomized Clinical Trial. Blood.

[3] Palumbo A, Cavallo F, Gay F (2014). Autologous transplantation and maintenance therapy in multiple myeloma. N Engl J Med..

[4] Tiede A, Priesack J, Werwitzke S (2008). Diagnostic workup of patients with acquired von Willebrand syndrome: a retrospective single-centre cohort study. J Thromb Haemost.

[5] Tiede A, Rand JH, Budde U (2011). How I treat the acquired von Willebrand syndrome. Blood.

[6] Callaghan MU, Wong TE, Federici AB (2013). Treatment of acquired von Willebrand syndrome in childhood. Blood.

[7] Greipp PR, San Miguel J, Durie BG (2005). International staging system for multiple myeloma. J Clin Oncol.

[8] Einsele H, Engelhardt M, Tapprich C (2017). Phase II study of bortezomib, cyclophosphamide and dexamethasone as induction therapy in multiple myeloma: DSMM XI trial. Br J Haematol.

[9] Fruehauf S, Klaus J, Huesing J (2007). Efficient mobilization of peripheral blood stem cells following CAD chemotherapy and a single dose of pegylated G-CSF in patients with multiple myeloma. Bone Marrow Transplant.

[10] Durie BGM, Harousseau JL, Miguel JS (2006). International uniform response criteria for multiple myeloma. Leukemia.

[11] Federici AB, Stabile F, Castaman G (1998). Treatment of acquired von Willebrand syndrome in patients with monoclonal gammopathy of uncertain significance: comparison of three different therapeutic approaches. Blood.

[12] Katagiri S, Akahane D, Amano K (2016). Long-term remission of acquired von Willebrand syndrome associated with multiple myeloma using bortezomib and dexamethasone therapy. Haemophilia.

[13] Engelen ET, van Galen KPM, Schutgens RE (2015). Thalidomide for treatment of gastrointestinal bleedings due to angiodysplasia: A case report in acquired von Willebrand syndrome and review of the literature. Haemophilia.

[14] Lavin M, Brophy TM, Rawley O (2016). Lenalidomide as a novel treatment for refractory acquired von Willebrand syndrome associated with monoclonal gammopathy. J Thromb Haemost.

[15] Djunic I, Elezovic I, Ilic V (2014). The effect of paraprotein on platelet aggregation. J Clin Lab Anal.

